# Gene trapping identifies chloride channel 4 as a novel inducer of colon cancer cell migration, invasion and metastases

**DOI:** 10.1038/sj.bjc.6605536

**Published:** 2010-01-19

**Authors:** T Ishiguro, H Avila, S-Y Lin, T Nakamura, M Yamamoto, D D Boyd

**Affiliations:** 1 Cancer Biology Department, MD Anderson Cancer Center, Houston, TX, USA; 2 Systems Biology Department, MD Anderson Cancer Center, Houston, TX, USA; 3 Surgical Oncology Department, Hokkaido University Graduate School of Medicine, Sapporo, Japan; 4 Second Department of Surgery, Hamamatsu University School of Medicine, Hamanatsu, Japan

**Keywords:** colorectal cancer, enhanced retroviral mutagenesis, chloride channel 4, migration, invasion, microenvironment

## Abstract

**Background:**

To date, there are few reports on gene products contributing to colon cancer progression.

**Methods:**

We used a gene trap comprised of an enhanced retroviral mutagen (ERM) cassette that includes a tetracycline-responsive promoter upstream of a haemagglutinin (HA) tag and a splice donor site. Integration of the ERM within an endogenous gene yields a tetracycline-regulated HA-tagged transcript. We transduced RKO colon cancer cells expressing a tetracycline *trans*-activator-off with the ERM-encoding retrovirus and screened for enhanced migration.

**Results:**

One clone showed fivefold enhanced migration with tetracycline withdrawal. Rapid amplification of cDNA ends identified the trapped gene as the chloride channel 4 (*CLCN4*) exchanger. Stable expression of a CLCN4 cDNA enhanced motility, whereas cells knocked down or null for this transcript showed reduced migration/invasion. CLCN4-overexpressing RKO colon cancer cells were more resistant than controls to proton load-induced cytotoxicity, consistent with the H^+^-extruding function of this antiporter. Intra-splenic delivery of RKO-CLCN4 transfectants, but not controls, yielded liver metastases, and transcript levels were higher in colon cancer metastases to the liver when compared with primary tumours.

**Conclusions:**

CLCN4 is a novel driver of colon cancer progression.

Sporadic colorectal cancer, afflicting 145 000 persons per year in the United States (de la Chapelle, 2004), largely reflects aberrantly activated pathways, leading to unrestrained growth. In the Wnt pathway, adenomatous polyposis coli truncations stabilise *β*-catenin, leading to the *trans*-activation of target genes causal for growth (Heyer *et al*, 1999). Mutation-activated K-Ras (Aktas *et al*, 1997) also promotes tumour growth, whereas mutation of the type II transforming growth factor *β* (TGF-*β*) receptor gene (*TGF-β RII*) generating a truncated protein is incapable of restraining growth (Derynck *et al*, 2001). Mutation inactivation of the p53 tumour suppressor renders damaged cells unable to arrest for DNA repair, leading to the mutation of key genes crucial to tumour development (Levine, 1997). Emerging studies also indicate that the heterogeneity of this disease probably also involves the contribution of multiple other gene products (Tomlinson *et al*, 2007; Wood *et al*, 2007) acting in various combinations to promote cancer development and progression.

Although the aforementioned observations have provided invaluable insight into the molecular mechanisms responsible for colorectal cancer development, there have been relatively few studies that identify gene products contributing to colon cancer cell migration, invasion and metastases (Cajot *et al*, 1997; Guan *et al*, 2000; Minard *et al*, 2005; Samuels *et al*, 2005). As one example, *Drg-1*, identified by differential display, is downregulated in its expression in metastatic colon cancer and its forced overexpression reduces *in vitro* invasion of colon cancer, these findings leading the authors to propose *Drg-1* as a metastatic suppressor (Guan *et al*, 2000). Similarly, expression of motility-related protein (MRP1/CD9) is highly repressed in metastases compared with primary colorectal tumours, and anti-MRP1/CD9 antibodies abrogate tumour cell migration (Cajot *et al*, 1997). Conversely, the *PIK3CA* gene is mutated in ∼30% of colorectal cancers (Samuels *et al*, 2004) and these alterations generate a protein that facilitates tumour invasion (Samuels *et al*, 2005). Considering the paucity of studies to identify genes regulatory for colon cancer cell migration/invasion, we undertook an unbiased, genome-wide approach to discover novel genes stimulatory for colon cancer cell migration. Towards this end, we used a gene-trap method (Liu *et al*, 2000; Lin and Elledge, 2003) (see Figure 1 for strategy) using a retroviral enhanced retroviral mutagen (ERM) cassette. The ERM includes a haemagglutinin (HA) tag and a splice donor site under the control of a tetracycline-responsive promoter. On retroviral transduction of tetracycline *trans-*activator-expressing cells, if the ERM is integrated intra-genically, the endogenous gene is brought under the control of the tetracycline-responsive promoter and the spliced ‘gene-trapped’ transcript can be identified by the HA tag sequence using rapid amplification of cDNA ends (3′-RACE). Using this gene-trap system, we report herein the identification of chloride channel 4 (*CLCN4*) as a novel gene product that enhances colon cancer migration and metastases.

## Materials and methods

### Cell lines

The RKO and HCT 116 colon cancer cell lines were all established from resected primary colon tumours (Brattain *et al*, 1981, 1984). LS174 cells were derived from a colon adenocarcinoma that had spread to the pericolonic fibroadipose tissue (Kahan *et al*, 1976). *ClCN4* wild-type and null mouse fibroblasts have been described elsewhere (Mohammad-Panah *et al*, 2009).

### Design of modified ERM vectors

Enhanced retroviral mutagen vectors are pBabe-puro based. The ERM cassette, comprising of a tetracycline-responsive promoter, an HA Tag and an artificial splice donor (AAGGTAAGT), was cloned into the *Nhe*I site at the 3′ long terminal repeat sequence. A set of three ERM vectors were constructed in which the ERM Tags were in all three possible reading frames: RF1, RF2 and RF3. All plasmids (RF1, RF2, RF3, PCGP, VSV, tetracycline *trans*-activator (tTA)) were propagated in *Escherichia coli* DH5*α* cells.

### Construction of CLCN4 expression constructs

Full-length CLCN4 cDNA was purchased from the American Type Culture Collection (ATCC-X77197; cat. no.8427856) and subcloned into the BlueScript vector. The CLCN4 cDNA sequence was then subcloned into the multicloning site of pcDNA3.1(−) using *Eco*RI and *Bam*HI enzymes to generate the CLCN4 pcDNA 3.1 construct. A partial cDNA sequence was then generated by PCR to include the *Nsi*I site located within the CLCN4 sequence and extending to the 3′ end of the cDNA. The 3′ primer was designed to include an HA sequence, followed by a stop codon and a *Bam*HI site. The CLCN4 pcDNA 3.1 construct was digested with *Nsi*I and *Bam*HI to generate a 5′ fragment of the cDNA sequence. The 5′ and HA-tagged 3′ CLCN4 sequences digested with *Nsi*I and *Bam*HI were then ligated. To generate truncated CLCN4 cDNA, the CLCN4 pcDNA 3.1 construct was digested with *Xba*I to delete the 5′ coding sequence (restriction sites are located in the vector multicloning site and there is a unique site in the cDNA sequence) and autoligated, thus engineering a cDNA corresponding to the trapped endogenous gene.

### Virus production

293T cells were maintained in Dulbecco’s modified Eagle’s medium containing 10% (v/v) foetal calf serum (FCS). 293T cells (9 × 10^6^) were seeded in 10 ml of 10% FCS for 12 h before transfection. HEPES-buffered saline solution (pH 7.05; 1 ml) was added to an equal volume of DNA/CaCl_2_ solution containing 8 *μ*g of transfer vector construct, 6 *μ*g of gag/pol packaging plasmid and 4 *μ*g of VSVg envelope plasmid, and the resulting solution was immediately added to the above medium. The cells were then returned to the 32°C incubator (5% CO_2_) for 8 h. Subsequently, the medium was changed to 10 ml of fresh 10% FCS, and 48 h later, the medium was removed, centrifuged at 500 × *g* for 5 min and filtered through a 0.45 *μ*m filter. The virus supernatant was stored at −80°C in aliquots for subsequent infection of cancer cells in MOI (multiplicity of infection) of 1 : 1.

### Cancer cells infection with ERM retrovirus

Colon cancer cells were maintained in McCoy’s 5A medium supplemented with 10% foetal bovine serum. Cancer cells were plated in 100 mm plates (5 × 10^5^ cells per plate) and infected with the virus supernatant of the tTA in the presence of 4 *μ*g/ml polybrene. The medium was changed to McCoy’s 5A/10% CS 5 h later. Cells were harvested 48 h later and selected in G418. Cancer cells expressing the tetracycline-responsive promoter *trans*-activator (tTA) were infected with ERM virus supernatants as described above, except for puromycin selection (1 *μ*g/ml), and then expanded for further analysis.

To determine the viral titre, antibiotic selections (G418 for tTA and puromycin for RF1, 2, 3) were performed as above, except that, at 48 h after infection, the cells were split at a 1 : 100 ratio into selection medium, fed every 3 days and the colonies were counted 10 days later. In determining the G418-resistance titre, the number of colonies was divided by four to account for two cell doublings. Transformed colonies in selected areas were enumerated 10 days after infection.

### Migration Assays

Unless specified otherwise, cells (5 × 10^4^) were suspended in 0.1 ml of 0.1% albumin-containing culture medium and dispensed into the upper chambers of Transwells (8 *μ*m pore size), with 0.6 ml of 10% FBS-containing medium in the lower chamber. After 6–8 h, cells on the upper surface were removed with a cotton swab, and cells migrating to the lower membrane surface were either detached with trypsin or stained with haematoxylin/eosin or Hema-Diff.

### Identification of target genes

To identify trapped genes, total RNA was extracted from expanded clones using RNeasy Mini Kits (Qiagen, Valencia, CA, USA). Reverse transcription (RT) was performed with a primer RT-1 (5′-GCAAATACGACTCACTATAGGGATCCNNNN(GC)ACG-3′) containing the random sequence NNNN and using the Superscript III kit (Invitrogen, Carlsbad, CA, USA). The 5′ end of the primer corresponds to the T7 primer sequence. The cDNA was then PCR amplified using an ERM-specific primer (5′-GACACCGGGACCGATCCAG-3′) and the T7 primer (T7-2, 5′-GCAAATACGACTCACTATAGGGATC-3′) using AccuTaq DNA polymerase (Invitrogen). The PCR products were gel purified, directly sequenced and nucleotide sequences were searched against the human non-redundant Genebank and expressed sequence tag (EST) databases using the Basic Local Alignment Search Tool (Blast, http://blast.ncbi.nlm.nih.gov/Blast.cgi).

### Reverse transcription-Polymerase Chain Reaction

The RNA was isolated using the RNAeasy Mini Kit (Qiagen) according to the manufacturer's instructions. Reverse transcription was performed from 2 *μ*g of RNA using SuperscriptII (Invitrogen). The cDNA was then PCR amplified using Taq DNA polymerase (PGC Scientific, Garner, NC, USA). Primers used for CLCN4 were as follows: forward 5′-GGACGAGTTTACTCACCGCA-3′ (in exon 11) and reverse 5′-GGTCCTGGTTTGCCATCTGG-3′ (in exon 13). The Tm values were 58 and 61°C, respectively, and 35 cycles of PCR were used.

### Quantitative PCR

The RNA was extracted using Trizol as per the manufacturer’s instructions (Invitrogen) and reverse transcribed at 37°C for 2 h using 2 *μ*g RNA as input. Quantitation was performed using a 7500 real-time PCR system (Applied Biosystems, Foster City, CA, USA) and the Taqman gene expression assay for CLCN4 (Applied Biosystems assay; ID-HS001565411_m1; cat. no. 70771B2) with primers spanning the exon 4–5 boundary. GAPDH transcript levels were used as an internal control (Applied Biosystems cat. no. 4333764F). Results were calculated using the comparative threshold cycle (C*t*) method of relative quantitation, as described by the manufacturer.

### Cell invasion assays

Cell invasion assays were carried out using Matrigel-precoated Transwell chambers (8 *μ*m pore size). Unless indicated otherwise, cells (5 × 10^5^) suspended in 0.125 ml of serum-free medium containing 0.5% BSA were added to each upper well and cells in 0.75 ml of medium containing 10 or 20% FBS were added to the corresponding lower well. After 16–22 h, non-invasive cells were removed from the upper aspect of the membrane by scrubbing with a cotton swab and cells on the lower aspect were either recovered with trypsin and enumerated or fixed and stained using Hema-Diff, as instructed by the manufacturer (StatLab, McKinney, TX, USA).

### Scratch/wound migration assay

Near confluent cells were scratch wounded with a sterile pipette tip, the monolayer changed to fresh medium containing 1% FBS and images captured at 0 and 24 h. Migration distances were determined using a superimposed digital 5 mm scale bar.

### siRNA knockdowns

The following CLCN-4-targeting and non-targeting siRNAs from Ambion (Austin, TX, USA) were used: no. 1 (cat no. 16708A SiRNA ID no. 7181; sense GGUGGCAAUUAUUUUCAGAtt; antisense UCUGAAAAUAAUUGCCACCtt) no. 2 (cat no. 16708A SiRNA ID no. 104393; sense GGCUGAUGUUUGUAACUUAtt; antisense UAAGUUACAAACAUCAGCCtt); no. 3 (cat no. 16708A SiRNA ID no. 146048; sense GGACGAGUUUACUCACCGCtt; antisense GCGGUGAGUAAACUCGUCCtt). The non-targeting control was the silencer negative control SiRNA (cat no. 4635).

### DNA transfection

Lipofectamine 2000 was used for delivery of plasmid DNA and siRNA into the colon cancer cells as per the manufacturer's instructions (Invitrogen).

### Transduction of colon cancer cells with an shRNA targeting CLCN4

LS174 cells were transduced with CLCN4 lentiviral particles encoding an anti-CLCN4 shRNA (SH-006152-03-10) or a non-targeting shRNA (S-005000-01) (Lentigen, Gaithersburg, MD, USA) using 10 *μ*g ml^−1^ polybrene at 10 MOI. Cells were puromycin (10 *μ*g ml^−1^) selected and fluorescence activated cell sorted for high EGFP expression. The recovered cells were expanded.

### Statistical analysis

Data were expressed as the mean±standard error (s.e.). Unless indicated otherwise, an unpaired *t*-test was used to determine whether differences were statistically different using GraphPad Prism software (v 5.02, La Jolla, CA, USA). A *P*-value of <0.05 was considered to be statistically significant.

### Experimental metastases assay

Cells (10^6^) were inoculated into the spleen of nude mice as described previously (Jessup *et al*, 1989) and, after 6 weeks, livers were examined macroscopically for metastatic disease.

### Western blotting

Cells were lysed with a buffer (15 mM NaCl, 10 mM Tris (pH 7.4), 1% TritonX-100, 0.5% Igepal (Sigma I3021, St Louis, MO, USA), 1 mM EDTA, 1 mM EGTA (pH 7.4), 200 *μ*M PMSF and protease inhibitor cocktail (Roche 11836153001, Indianapolis, IN, USA)). Samples (20 *μ*g) were run in an 8% gel for 1 h at 40 mA and proteins transferred for 2 h at 100 V to a PDF membrane. Protein detection was performed by sequential hybridisation with a 1 : 500 dilution of an anti-HA rabbit antibody (Invitrogen cat no. 715500) and 1 : 2500 dilution of a goat anti-rabbit-HRP-conjugated antibody.

## Results

### Gene trapping of *CLCN4*

**Figure 2:**
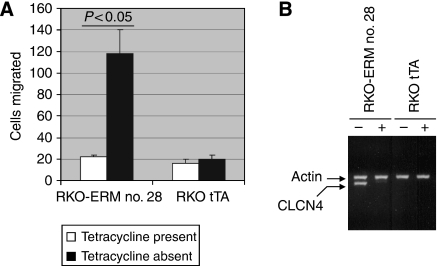
Trapping of a gene stimulatory for cell migration. (**A**) The indicated cells (5 × 10^4^) grown for 72 h with or without tetracycline (2 *μ*g ml^−1^) were added to the upper chamber of transwell inserts. The lower chamber contained 10% FBS. After 6 h, cells migrating to the lower surface of the membrane were stained, and the number of cells was counted in five fields and averaged (±s.e.). Data are representative of at least three experiments. (**B**) The indicated cells were treated with or without 2 *μ*g ml^−1^ tetracycline for 72 h, RNA was extracted and subjected to RT–PCR (35 cycles) using primers specific for CLCN4 and actin.

We first infected RKO colon cancer cells with a retrovirus encoding a tTA expression plasmid and isolated tetracycline-responsive clones using a tTA-responsive luciferase reporter. One clone, showing a fourfold induction in response to tetracycline removal (data not shown), was used for subsequent gene trapping. We infected these cells with the ERM retrovirus to generate a library of gene-trapped cells, and transwell motility assays were performed thrice sequentially to select for cells with enhanced migration. Accordingly, 97 independent clones showing augmented migration were isolated and, of these, 13 showed increased migration in the absence, but not in the presence, of tetracycline. One clone (RKO-ERM no. 28) manifested a robust (∼sixfold, *P*<0.05) increase in migration on tetracycline removal (Figure 2A), whereas the RKO cells expressing only tTA showed no modulation on tetracycline withdrawal. We isolated total RNA from RKO-ERM no. 28 cells and performed 3′ RACE with the ERM tag sequence to identify the trapped endogenous gene. A homology search revealed the trapped gene to be *CLCN4*. Reverse transcription-PCR showed that tetracycline removal caused a robust induction of a transcript using primers corresponding to the CLCN4 message for the RKO-ERM no. 28 clone but not with the tTA-expressing RKO cells (Figure 2B). The 3′ RACE indicated that the CLCN4 gene was trapped at exon 7. Analysis of the predicted protein sequence by Ensembl (www.ensembl.org) and the SMART protein prediction algorithm (http://smart.embl-heidelberg.de) indicated four Prints domains (http://www.bioinf.man.ac.uk/ dbbrowser/PRINTS/index.php) constituting the CLCN4 encoded by exons 7–9, as well as the two cystathionine *β*-synthase motifs encoded by exons 8 and 9. In addition, four transmembrane-spanning segments encoded by exons 7 and 8 were present in the gene-trapped product.

We pursued CLCN4 for several reasons. First, the gene is encoded at Xp22.3, a chromosomal region that shows gain in 16% of colon cancer patients (http://130.60.44.174/progenetix/). Second, CLCN4 is an H^+^/Cl^−^ exchanger (in which the movement of Cl^−^ in one direction is coupled to the movement of protons in the opposite direction) and chloride fluxes have been shown to modulate cell migration (Ransom *et al*, 2001). Third, persistent metabolism of glucose to lactate by tumour cells (adaptative response to hypoxia) (Gatenby and Gillies, 2004) requires a mechanism for proton extrusion to prevent acidosis-induced cytotoxicity.

### Validation of the gene-trapped CLCN4 as a regulator of colon cancer cell migration

**Figure 3:**
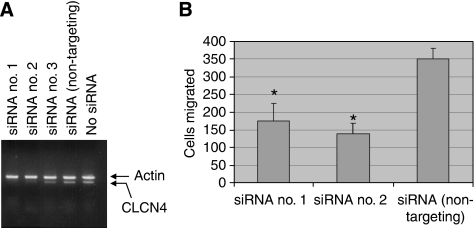
Knockdown of gene-trapped *CLCN4* counters tumour cell migration. RKO-ERM no. 28 cells, induced for *CLCN4* expression by tetracycline withdrawal, were transiently transfected with a CLCN4-targeting (siRNA no. 1, 2, 3) or a non-targeting siRNA using lipofectamine 2000. After 48 h, cells were either RNA extracted for semi-quantitation of CLCN4 transcript levels by RT–PCR (35 cycles) using primers located in exons 11 and 13 (**A**) or 5 × 10^4^ cells were analysed for cell migration (**B**) as per Figure 2. Data in **B** represent mean±s.e. values for triplicate determinations. asterisk indicates, *P*<0.05 compared with that of the non-targeting siRNA.

**Figure 4:**
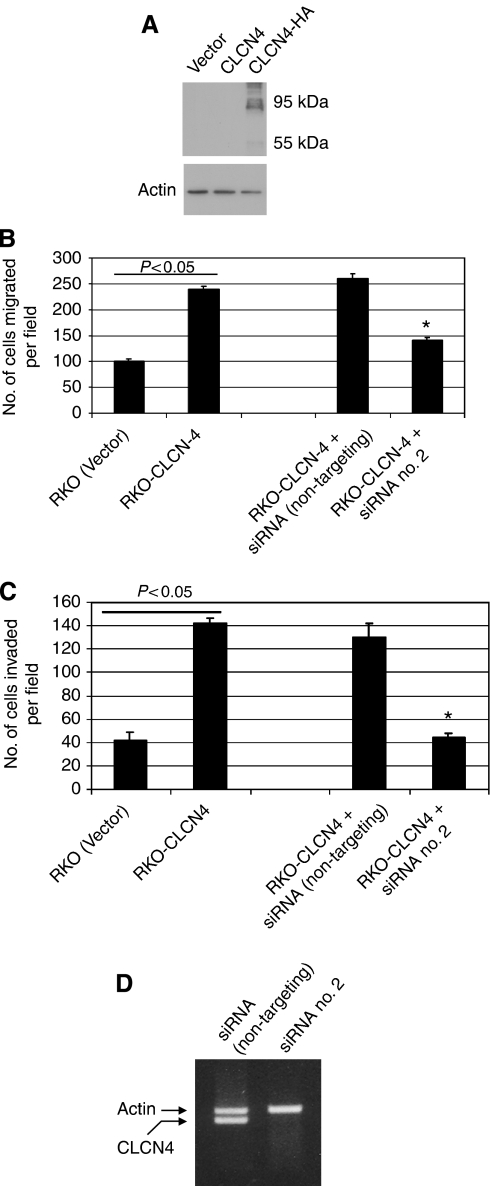
Expression of a full-length CLCN4 induces colon cancer cell migration. (**A**) A pool of RKO colon cancer cells stably expressing the indicated constructs were lysed and 20 *μ*g protein was analysed by western blotting using a rabbit anti-HA tag antibody (Invitrogen cat no. 715500), followed by an HRP-coupled goat anti-rabbit IgG. Immunoreactive proteins were visualised by enhanced chemiluminescence. Size markers are indicated. (**B**) A pool of RKO clones overexpressing wild-type CLCN4 (RKO-CLCN4) or the empty vector (RKO (Vector)) were assayed for migration as described in Figure 2 and using 10% FBS as chemoattractant. (**C**) *In vitro* invasion was as described for the migration assays, with the exception that a Matrigel-coated filter was used and cells traversing the coated filter were enumerated 16 h later. The experiment was repeated three times and data are shown as average±s.e values. For (**B**) and (**C**), an asterisk indicates *P*<0.05, in comparison with the RKO-CLCN4 data. (**D**) RT–PCR was carried out for CLCN4 expression as described in Figure 3.

It remained a formal possibility that the tetracycline-responsive promoter was modulating a gene(s) neighbouring *CLCN4* that was causal for the altered migration. Further, as the endogenous *CLCN4* gene was trapped at exon 7 (determined by 3′ RACE), the expressed truncated protein (Supplementary Data 1), while retaining much of the chloride channel structure, might invoke a biological effect unrelated to its physiological role. Thus, to validate the gene-trapped CLCN4 as an inducer of tumour cell migration, the following experiments were performed. First, we used a siRNA strategy to determine whether silencing of the endogenous gene (induced by tetracycline withdrawal) in RKO-ERM no. 28 cells would counter cell migration. Of three independent siRNAs tested, two (siRNA no. 1 and no. 2) were effective in countering CLCN4 expression in RKO-ERM no. 28 cells (Figure 3A) induced by tetracycline withdrawal. Moreover, transient transfection of CLCN4-induced RKO-ERM no. 28 cells with siRNA no. 1 or no. 2 reduced (*P*<0.05) cell migration (Figure 3B) when compared with the non-targeting siRNA. Second, we determined whether the expression of a full-length CLCN4 cDNA would reiterate the effects on migration of the endogenously trapped CLCN4 gene. Towards this end, an HA-tagged full-length CLCN4 cDNA was subcloned into the pIRES-EGFP2 bicistronic expression construct and stably transfected into RKO cells. Cells were then selected with G418, and the GFP-positive pooled population was analysed by western blotting using an anti-HA antibody (Figure 4A). As expected, we detected the expression of the full-length HA-tagged CLCN4 protein at the predicted size of 95 kDa, but not non-HA-tagged CLCN4. More importantly, RKO cells expressing full-length CLCN4 cDNA showed enhanced migration (*P*<0.05) in two independent assays (Figure 4B, Supplementary Data 2) and *in vitro* invasion (Figure 4C), compared with transfectants harbouring the empty vector. Monolayer proliferation was unaffected by CLCN4 expression (data not shown). Moreover, transient transfection of the CLCN4 siRNA no. 2 countered (*P*<0.05) this increased migration/invasion, concomitant (Figure 4B, C) with silencing of the exogenous construct (Figure 4D).

**Figure 5:**
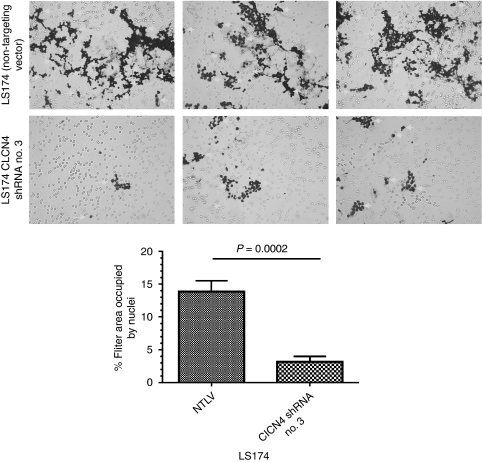
Knockdown of CLCN4 reduces migration of colon cancer cells. The indicated cells (100 000) were seeded in triplicate in 10% FBS on porous filters (8 *μ*m). The bottom well contained 20% FBS in McCoy’s 5A medium. After 6 h, cells were scrubbed from the top aspect of the filter, cells on the bottom aspect were fixed and stained with Hema-Diff, as instructed by the manufacturer. Images were taken using a Nikon Microphot-FXA microscope (Nikon, Melville, NY, USA) at × 100 magnification, digitised and the nuclear area was captured with Corel Photo-Paint (v.11) using the colour mask pick tool feature to identify the nuclei. This image was captured and the nuclear area was quantified using ImageJ 1.42 software (http://rsb.info.nih.gov/ij/). Data are shown as the average percentage area of the filter occupied with nuclei±s.d. values of at least four independent fields. Nuclei are marked with white arrows. Statistical analysis was performed using an unpaired *t*-test and GraphPad Prism software version 5.02.

**Figure 6:**
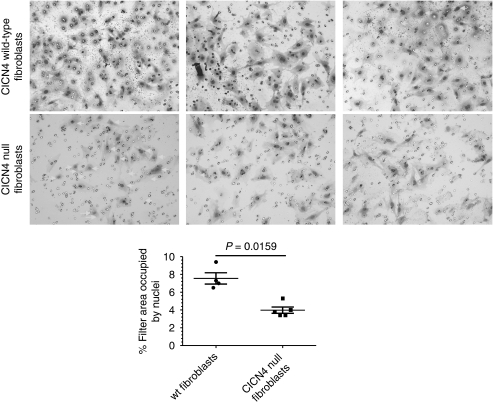
Reduced invasiveness of null *CLCN4* fibroblasts. These assays were as described for Figure 5, but with the following exceptions. The indicated cells (20 000) were seeded in triplicate on Matrigel-coated porous filters (8 *μ*m) and, after 22 h, were assayed and quantified for invasion. Data are shown as the average percentage area of the filter occupied with nuclei±s.d. values of at least four independent fields. Nuclei are marked with white arrows. Statistical analysis was performed using a Mann–Whitney *U-*Test.

We then investigated whether knockdown of the CLCN4 transcript would interfere with cell migration and invasion. Towards this end, we transduced LS174 colon cancer cells with lentivirus particles bearing a CLCN4 shRNA or, as a control, a non-targeting shRNA. After selection of a puromycin-resistant population, the cells bearing CLCN4-targeting shRNA showed a 65–70% reduction in transcript levels compared with their counterparts expressing the non-targeting shRNA by quantitative PCR. We then assayed these cells for their migratory capacity through a porous filter. To remove investigator bias, images were digitised, stained nuclei were ‘captured’ by Corel Photo-Paint (Corel, Ottawa, Ontario, Canada) and quantified by ImageJ software (http://rsb.info.nih.gov/ij/). In these assays, the LS174 cells bearing CLCN4 shRNA were less migratory (Figure 5) compared with control cells harbouring the non-targeting shRNA (13.8±1.7 *vs* 3.2±0.8%, filter area masked with nuclei), this difference being statistically significant (*P*=0.0002). We also compared the ability of mouse fibroblasts wild type and null for *CLCN4* for *in vitro* invasiveness. Cells were seeded onto Matrigel-coated porous filters, and after 22 h, assayed for their ability to penetrate the extracellular matrix-coated filter by staining cells on the lower aspect of the membrane. We found that *CLCN4* wild-type cells showed about a twofold higher rate of invasion (area of filter occupied with nuclei=7.5±1.3 *vs* 3.9±0.8, *P*=0.0159) compared with their null counterparts (Figure 6). Thus, we conclude that the wild-type *CLCN4* does indeed contribute to cell migration.

**Figure 7:**
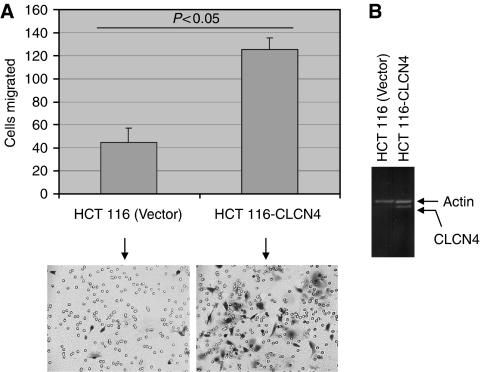
CLCN4 overexpression induces migration of an independent colon cancer cell line. (**A**) Pools of HCT 116 cells stably overexpressing the full-length CLCN4 cDNA (HCT 116-CLCN4) or the empty pcDNA 3.1 vector (HCT 116 (Vector)) were assayed for migration as described for Figure 2. Arrows indicate migrated cells. Data are mean±s.e. values of three separate determinations. (**B**) CLCN4 overexpression was verified by RT–PCR (35 cycles) using primers located in exons 11 and 13.

To determine the generality of CLCN4 in augmenting cell migration, a third colon cancer cell line (HCT 116) was stably transfected with full-length CLCN4 and assayed for cell migration. Similar to RKO colon cancer cells, CLCN4 overexpression (Figure 7B) also yielded an enhanced migration (*P*<0.05) of HCT 116 cells (Figure 7A). Thus, the modulatory effect of CLCN4 on tumour cell migration is not restricted to RKO and LS174 colon cancer cell lines.

### Elevated CLCN4 induces liver metastases

**Figure 8:**
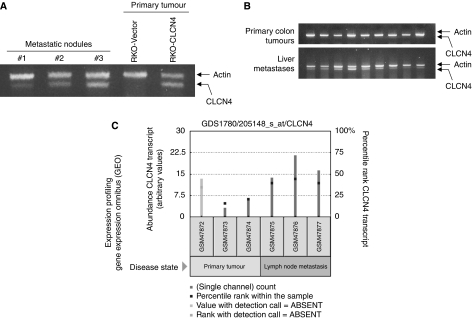
CLCN4 stimulates experimental metastases and is elevated in expression in metastases to the liver/lymph nodes. (**A**) Pooled RKO clones overexpressing full-length CLCN4 cDNA (RKO-CLCN4) or the empty vector (RKO-vector) were injected (10^6^ cells) into the spleen of groups of 10 nude mice. After 6 weeks, the mice were killed and liver tissues were examined macroscopically for metastatic tumour nodules or subjected to RT–PCR for CLCN4. (**B**) RNA extracted from a series of primary colon tumours and colon cancer metastases to the liver was subjected to RT–PCR for CLCN4 expression as per Figure 7. (**C**) Query of the Gene Expression Omnibus database (http://www.ncbi.nlm.nih.gov/projects/geo/) showing increased CLCN4 expression in colon cancer metastases to the lymph nodes compared with synchronous primary tumours. Squares within each bar represent the percentile rank of the CLCN4 transcript for all transcripts within the sample (right axis). Left axis shows the relative scale for the measured level of abundance of the CLCN4 transcript (arbitrary values). The faded bar represents an absent detection call (Affymetrix, Santa Clara, CA, USA).

Owing to the fact that increased CLCN4 expression augmented *in vitro* migration and invasion, we hypothesised that CLCN4 would increase the metastatic ability of colon cancer cells. To answer this question, RKO colon cancer cells overexpressing CLCN4, or the vector alone, were injected into the spleen of nude mice. After 6 weeks, the livers were examined macroscopically for metastatic nodules. Liver metastases was evident in 5 of 10 mice receiving CLCN4-overexpressing RKO cells, whereas no metastatic nodules were detected in vector controls. We verified continued overexpression of CLCN4 in colon cancer metastases to the liver (Figure 8A) by RT–PCR.

If CLCN4 promotes tumour spread, a high level of expression would be predicted in liver metastases in colon cancer patients. We therefore assayed CLCN4 transcript levels in a small series of resected human primary tumours and liver metastases. We observed a clear enrichment of CLCN4 expression in colon cancer metastases to the liver for all nine patients over that evident with primary tumours (Figure 8B). Although we cannot rule out the possibility of a CLCN4 signal from the liver itself (Jentsch, 2008), the tumour cellularity of the metastatic lesions was estimated to be >80%, making this possibility less likely. Nevertheless, to eliminate investigator bias, we also queried the Gene Expression Omnibus (GEO, http://www.ncbi.nlm.nih.gov/projects/geo/) expression profiling database for CLCN4 overexpression in colon cancer metastases. An elevated CLCN4 transcript level was again evident in colon cancer metastases to the lymph nodes compared with expression in the synchronous primary colon tumours (Figure 8C). Note the absence of detection call for the GSM47872 primary tumour sample. Taken together, these data suggest that CLCN4 stimulates colon cancer progression.

### CLCN4 expression protects against acid-induced cytotoxicity

**Figure 9:**
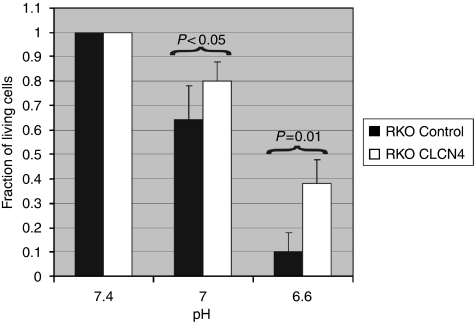
CLCN4-overexpressing colon cancer cells are more resistant to acid-induced cytotoxicity. Pooled RKO clones overexpressing full-length CLCN4 cDNA (RKO-CLCN4) or the empty vector (RKO-control) were cultured for 72 h at the specified pH (using a HEPES-buffered medium with hydrochloric acid/sodium hydroxide) and assayed for viability using MTT. Data are expressed relative to the value at pH 7.4. The experiment was undertaken three times. Statistical analysis was performed using an unpaired *t-*test.

Considering the fact that CLCN4, as a proton/Cl exchanger, extrudes protons from the cell against its electrochemical gradient (Picollo and Pusch, 2005), we argued that cells overexpressing the exchanger would be more resistant to the cytotoxic effect of an extracellular proton load. To answer this question, we cultured RKO cells overexpressing CLCN4, or the vector only, in an acidic medium akin to the pH evident in the tumour microenvironment (Gatenby and Gillies, 2004). Indeed, whereas over 35% of CLCN4-overexpressing RKO cells survived the reduced pH of 6.6, only 10% of vector controls tolerated the increased acidity (Figure 9), and this difference was statistically significantly (*P*=0.01).

## Discussion

Although there have been numerous investigations on genes contributing to colon cancer development, few studies have been undertaken to identify gene products implicated in colon cancer invasion/migration/metastases. Moreover, most of these studies have been empirical, focussing on ‘educated guesses’, with only a few using agnostic genome-wide studies (Cajot *et al*, 1997; Lombardi *et al*, 1999; Guan *et al*, 2000; Agrawal *et al*, 2002; Sordat *et al*, 2002). We describe herein gene trapping of the Cl^−^/H^+^ exchanger, CLCN4, as a novel promoter of colon cancer migration, invasion and metastases. Further, we show that CLCN4 expression is probably elevated in colon cancer metastases to the liver, our findings being consistent with the observation of increased mRNA levels in metastases to the lymph nodes as per a query of the GEO database. As the region of the genome (Xp22.3) harbouring CLCN4 shows gain/amplification in 16% of colon cancer patients, we presume that CLCN4 overexpression reflects, in part, increased gene dosage.

Although, to our knowledge, this is the first report of a causal role for CLCN4 in tumour progression, an earlier study (Soroceanu *et al*, 1999) is relevant for showing that pharmacological blockade of chloride channels was shown to reduce glioma cell migration and invasion into foetal rat brain aggregates. Unfortunately, the responsible chloride channel/exchanger driving migration/invasion was not identified in that investigation and, at present, at least 11 separate chloride exchangers/antiporters have been reported (Bustin *et al*, 2001; Jentsch, 2008). In contrast, the expression of two other chloride channels (the Ca^2+^-dependent chloride channels CLCA1 and CLCA2) was downregulated in 80% of colorectal tumours, although the biological significance of this repression (Bustin *et al*, 2001) remains to be determined. Other ion transporters, in addition to the aforementioned chloride channels/exchangers, have also been implicated in cancer cell migration/invasion. In this regard, silencing of MS4A12, a cell surface protein that controls Ca^2+^ flux into colon cells, attenuated cell migration and invasion of malignant intestinal cells (Koslowski *et al*, 2008). In addition, although implicated in tumour development rather than cell invasion/metastases, SLC5A8, a sodium symporter identified by a global search for genes that were aberrantly methylated at high frequency in human colon cancer and consequently repressed in expression (Li *et al*, 2003), was characterised as a candidate tumour suppressor. Finally, and reminiscent of our findings with CLCN4, expression of the ether a go-go (Eag1) potassium channel is linked with tumour metastases, in that its expression correlates with lymphatic node metastases and organ metastases (Ding *et al*, 2007). However, whether Eag1 is a driver of tumour spread or merely a passive bystander remains to be determined.

As to the mechanism by which CLCN4 promotes colon cancer migration, invasion and metastases, several possibilities can be entertained. As CLCN4 is a known Cl^−^/proton antiporter, the most likely mechanism relates to its role in ion exchange (Jentsch, 2008). Indeed, our unpublished findings that the general chloride channel inhibitor attenuated the effects of CLCN4 overexpression on tumour cell migration is in agreement with this contention, notwithstanding the caveat that this blocker targets other chloride channels as well. CLCN4 is displayed both at the cell surface and at endosomal membranes, possibly cycling between both compartments (Mohammad-Panah *et al*, 2002), and its ability to regulate fluxes in both protons and chloride ions may have ramifications both with respect to control of intracellular pH or the pH of the endosomal compartment (Mohammad-Panah *et al*, 2002). As discussed earlier, regulation of intracellular pH is critical in tumour cells in which proton accumulation is high because of incomplete glucose metabolism and a proton extrusion mechanism is necessary to maintain a neutral or near neutral intracellular pH (Gatenby and Gillies, 2004). In addition, regulation of pH in the endosomal compartment (Mohammad-Panah *et al*, 2002) could also have a bearing on the ability of CLCN4 to promote invasiveness, in that the acidification of large intracellular endosomal vesicles promotes proteolytic degradation of phagocytosed extracellular matrix (Montcourrier *et al*, 1994). Indeed, the quantitative presence of these vesicles correlates with cell migratory capacity, at least in models of breast cancer (Montcourrier *et al*, 1994). Alternatively, another possibility, although not mutually exclusive, is that modulation of salt levels as a consequence of chloride flux could yield water movement into/out of cells, thereby altering the cell shape and/or size so as to facilitate migration. In fact, such a mechanism was advanced to explain the invasive phenotype of cultured glioma cells initiated by volume-activated chloride fluxes (Ransom *et al*, 2001). Notwithstanding these aforementioned potential mechanism(s), it is also equally feasible that CLCN4 augments cancer progression through a mechanism unrelated to its role as a chloride/proton exchanger. Such an argument is not without precedent. Indeed, an in-frame deletion that abrogated anion exchange of the *DRA* transporter had no effect on the ability of the transporter to suppress tumour growth (Chapman *et al*, 2002). Furthermore, the fact that our endogenous CLCN4 gene trapped at exon 7, and therefore devoid of a part of the transmembrane-spanning region, nevertheless promoted cell migration might point to an additional mechanism(s).

In conclusion, using a gene-trap strategy, we identified *CLCN4* as a novel gene product that is stimulatory for colon cancer cell migration, invasion and metastases. These findings, and others (Soroceanu *et al*, 1999; Bustin *et al*, 2001; Chapman *et al*, 2002; Li *et al*, 2003; Ding *et al*, 2007), should direct attention to the importance of ion exchangers in tumour progression and could provide new avenues for managing patients at high risk for metastatic spread.

## Figures and Tables

**Figure 1:**
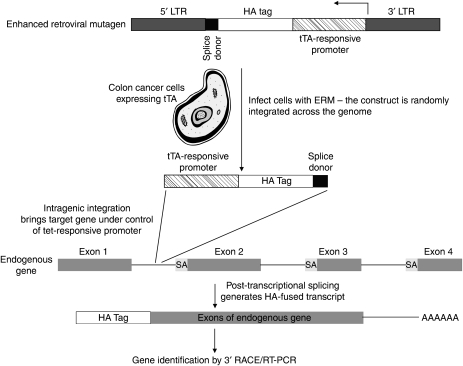
Schematic of gene-trapping experimental strategy. LTR, long terminal repeat; SA, splice acceptor; tTA, tetracycline-responsive *trans*-activator.
