# Supplementary figures and images for: Gene trapping identifies chloride channel 4 as a novel inducer of colon cancer cell migration, invasion and metastases
Source: Br J Cancer. 2010 Jan 19;102(4):774–82. doi: 10.1038/sj.bjc.6605536 (PMC2837579; doi:10.1038/sj.bjc.6605536)

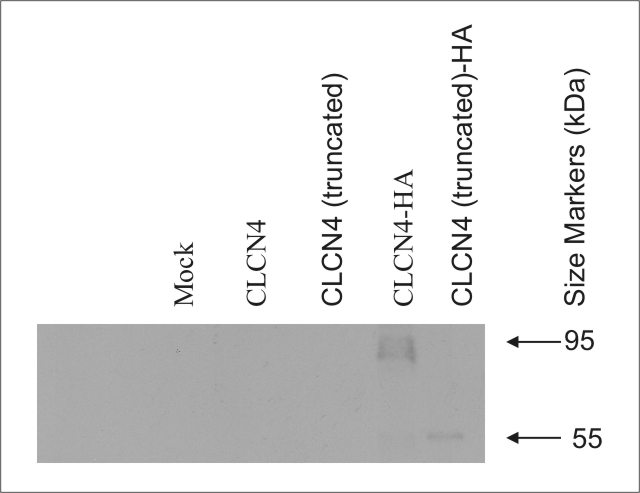

Supplement: Supplementary Figure 1 [file 6605536x1.tif]

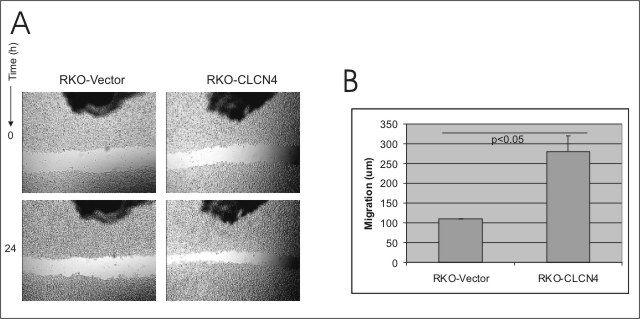

Supplement: Supplementary Figure 2 [file 6605536x2.tif]
