# Supplementary material for: Gene trapping identifies chloride channel 4 as a novel inducer of colon cancer cell migration, invasion and metastases
Source: Br J Cancer. 2010 Jan 19;102(4):774–82. doi: 10.1038/sj.bjc.6605536 (PMC2837579; doi:10.1038/sj.bjc.6605536)
Supplement: Supplementary Information [file 6605536x3.doc]

Supplementary Data 1. Expression of the truncated CLCN4 protein

RKO cells stably expressing the indicated constructs were lysed in a buffer containing 15 mM NaCl,10 mM Tris pH 7.4, 1% TritonX-100, 0.5% Igepal, 1 mM EDTA, 1 mM EGTA pH 7.4, 200 μM PMSF, and a protease inhibitor cocktail. Proteins (20 μg) were resolved by electrophoresis in an 8 % gel and transferred to a PDF membrane. After sequential hybridization with a (1: 500) dilution of an anti-HA rabbit antibody and 1:2,500 dilution of a goat anti-rabbit-HRP-conjugated antibody, immunoreactive proteins were detected by enhanced chemiluminescence.

Supplementary Data 2. CLCN4 over-expression enhances migration in scratch assays

Monolayers (>90 % confluent) of pooled RKO clones expressing the full length CLCN4 cDNA (RKO-CLCN4) or the empty vector (RKO-Vector) were scratch-wounded using a sterile pipette tip. Cultures in 1 % FBS were photographed at 0 and 24 h (Panel A). Distance measurements (Panel B) were made using a superimposed digital 5 mm bar scale. Data represent 3 determinations and are shown as mean + S.E. values.
